# Thermally induced crystallization in NbO_2_ thin films

**DOI:** 10.1038/srep34294

**Published:** 2016-09-29

**Authors:** Jiaming Zhang, Kate J. Norris, Gary Gibson, Dongxue Zhao, Katy Samuels, Minxian Max Zhang, J. Joshua Yang, Joonsuk Park, Robert Sinclair, Yoocharn Jeon, Zhiyong Li, R. Stanley Williams

**Affiliations:** 1 Hewlett Packard Labs, 1501 Page Mill Rd, Palo Alto, CA 94304, USA; 2 Department of Electrical and Computer Engineering, University of Massachusetts, Amherst, Massachusetts 01003, USA; 3 Department of Materials Science and Engineering, Stanford University, Stanford, CA 94305, USA

## Abstract

Niobium dioxide can exhibit negative differential resistance (NDR) in metal-insulator-metal (MIM) devices, which has recently attracted significant interest for its potential applications as a highly non-linear selector element in emerging nonvolatile memory (NVM) and as a locally-active element in neuromorphic circuits. In order to further understand the processing of this material system, we studied the effect of thermal annealing on a 15 nm thick NbO_2_ thin film sandwiched inside a nanoscale MIM device and compared it with 180 nm thick blanket NbO_x_ (x = 2 and 2.5) films deposited on a silicon dioxide surface as references. A systematic transmission electron microscope (TEM) study revealed a similar structural transition from amorphous to a distorted rutile structure in both cases, with a transition temperature of 700 °C for the NbO_2_ inside the MIM device and a slightly higher transition temperature of 750 °C for the reference NbO_2_ film. Quantitative composition analysis from electron energy loss spectroscopy (EELS) showed the stoichiometry of the nominal 15 nm NbO_2_ layer in the as-fabricated MIM device deviated from the target 1:2 ratio because of an interaction with the electrode materials, which was more prominent at elevated annealing temperature.

Niobium dioxide (NbO_2_) based metal-insulator-metal (MIM) structures are interesting candidates for electronic devices because they exhibit current-controlled threshold switching (TS) followed by an S-type negative differential resistance (NDR) in their current-voltage characteristic^1^,^2^,^3^,^4^,^5^,^6^,^7^. A recent report showed that NDR can occur in MIM structures for niobium oxide materials with different levels of crystallinity and stoichiometry^8^. An accurate compact dynamical model has been developed based on a thermal feedback mechanism, and numerical simulations agreed well with the electrothermal measurements of devices with different oxide thicknesses^8^. Thus, it is important to obtain insight into the structural changes of NbO_2_ and chemical interactions with electrodes during processing of MIM devices in order to control the material properties and to optimize the performance for electronic device applications.

NbO_2_ and related oxides such as VO_2_ are Mott insulators, which have been studied extensively because of their interesting thermally-induced insulator-metal transition (IMT)^9^,^10^,^11^. At room temperature, NbO_2_ possesses a tetragonal distorted rutile structure (*I*4_1_/*a*, *a* = 13.696 Å and *c* = 5.981 Å) with a band gap of 0.426 eV. At ~807 °C, NbO_2_ undergoes a reversible second-order phase transition resulting in a regular rutile structure (*P*4_2_/*mnm*, *a* = 4.846, *c* = 3.032)^12^. This structural change is accompanied by a change in the electrical properties, and NbO_2_ become a metallic conductor above 807 °C^13^,^14^. Although NbO_2_ is thermodynamically stable within a narrow composition range with negligible deviation from the exact stoichiometry, NbO_2-x_ sputtered films with well-controlled oxygen deficiency composition are reported to have superior TS^1^. A recent study using Transmission Electron Microscopy (TEM) combined with atom probe tomography showed local crystallization in amorphous NbO_2_ accompanied the TS behavior^15^. The present study focuses on the structural and chemical evolution of sputtered NbO_2_ films in two forms, a 180 nm thick film and a 15 nm thin film inside a nanoscale MIM device under thermal annealing in vacuum to emulate device processing conditions. High resolution transmission electron microscopy (HRTEM) and Scanning TEM associated electron energy loss spectroscopy (EELS) were performed to provide a systematic characterization of thermally induced crystallization in NbO_2_ films and the interaction between the NbO_2_ film and the electrodes.

## Results and Discussions

**Figure 1:**
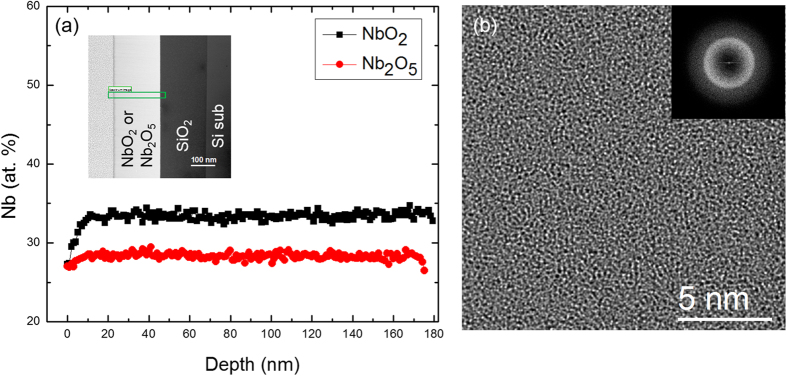
(**a**) Compositional profiles across NbO_2_ and Nb_2_O_5_ film (180 nm thick) from quantitative EELS analysis with cross-sectional STEM DF image in the inset. The STEM EELS spectrum image were collected with pixel size of 1 nm across the thin film (as shown in the rectangular area). The quantification were done by subtraction of pre-edge power law background and integrate Nb-M_4,5_ edge and O-K edge window. (**b**) HRTEM image of the as-prepared NbO_2_ film shows the amorphous microstructure and was confirmed by the diffuse ring in the fast Fourier transform in the inset.

Figure 1 shows the cross-sectional TEM and STEM/EELS analysis on the microstructure and chemical composition of the thicker NbO_2_ film (180 nm). The amorphous structure in the niobium oxide film is shown by the uniform contrast of the NbO_2_ layer in the STEM image in Fig. 1a and by an aperiodic structure in the HRTEM image in Fig. 1b. For compositional analysis, the deposited NbO_2_ film was compared to a magnetron sputtered stoichiometric Nb_2_O_5_ film. Two-dimensional STEM/EELS mapping were collected from the entire niobium oxide layer in the NbO_2_ and Nb_2_O_5_ specimens, as illustrated by the cross-sectional STEM dark field (DF) image in the inset. Each spectrum in the spectrum image (SI, i.e., the two-dimensional STEM/EELS maps with EELS spectra in the 3^rd^ dimension corresponding to each pixel) was quantified based on integration of the Nb-M and O-K edges, with a standard power-law background subtraction and cross section calculation using the Hartree-Slater model^16^,^17^. The Nb compositional profiles from the surface to the bottom of the oxide layer in both NbO_2_ and Nb_2_O_5_ were compared in Fig. 1a. Since Hartree-Slater calculations for electron scattering from M edges are less accurate than for K and L edges, an empirical correction factor between the cross-sections of Nb-M and O-K edges was used to calculate Nb:O ratio^17^,^18^. An average of Nb at.% of 28.6 ± 0.4% has been obtained across the entire Nb_2_O_5_ film (expected stoichiometric value is 28.5%). Similarly, the line profile in the films shows that the average of Nb at.% is 33.7 ± 0.4% in NbO_2_ (expected stoichiometric value is 33.3%), after removing a 10 nm surface with higher O composition due to exposure to the air after deposition. The EELS quantification results showed that both of the NbO_2_ and Nb_2_O_5_ films were close to the target composition. Figure 1b shows a HRTEM image of the NbO_2_ film with aperiodic structure, which is confirmed by the diffuse ring shown in the FFT in the inset. There was no detectable structural or chemical variation in the film based on the combined TEM images and EELS quantification analysis.

**Figure 2:**
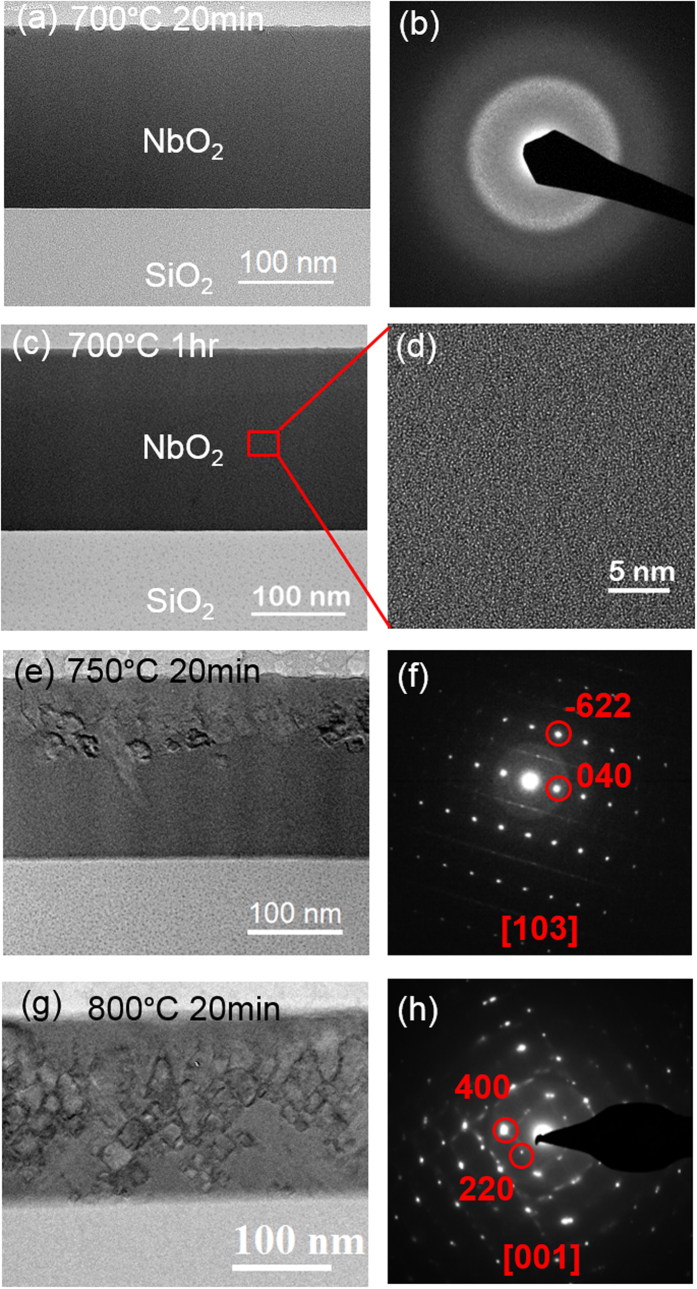
Microstructural evolution in NbO_2_ thick film as the function of annealing temperature. (**a**) BF cross-sectional TEM image and (**b**) SAED pattern at 700 °C; (**c**) BF image and (**d**) SAED pattern from NbO_2_ with distorted rutile phase along [103] at 750 °C; (**e**) BF image and (**f**) SAED pattern from NbO_2_ with distorted rutile phase along [001] at 800 °C.

To study the thermally induced crystallization behavior in NbO_2_, the as-fabricated 180 nm NbO_2_ film, as well as the 15 nm NbO_2_ film in the MIM device structure, were annealed at different temperatures up to 800 °C in vacuum to prevent further oxidization of NbO_2_^19^,^20^. Figure 2 shows the cross-sectional TEM images and selected area electron diffraction (SAED) patterns of the microstructural evolution in the 180 nm NbO_2_ film annealed between 700–800 °C. The amorphous structure in the 180 nm NbO_2_ film remained unchanged up to 700 °C. Figure 2a is the bright field (BF) TEM image showing that the structure of the NbO_2_ film annealed at 700 °C for 20 min has a uniform contrast with no visible crystalline grains. This is confirmed by the selected area diffraction pattern (SAED) in Fig. 2b, which shows only a diffuse ring pattern without any diffraction spots. Figure 2c is the BF image showing that the NbO_2_ film annealed at 700 °C for 1 hr still remains as amorphous structure. Figure 2d shows the high resolution TEM image of aperiodic structure in the NbO_2_ film at the atomic level. This suggests that 700 °C is certainly below the amorphous-to-crystalline transition temperature. A similar quantitative EELS analysis from NbO_2_ film annealed at 700 °C for 1 hr shows that the average of Nb at.% is 33.6 ± 0.5% in NbO_2_ film, consistent with that of the as-fabricated film. Figure 2e shows the upper part of the NbO_2_ film started to crystallize after annealing at 750 °C. The nucleation starts on the surface of the film due to the higher density of defects and larger oxygen content near the surface. The grain size ranged from 10 nm to 100 nm. A SAED pattern from one of the larger grain shows a crystalline zone pattern of [103] from tetragonal NbO_2_ (Fig. 2f). The weaker diffraction spots coexisting with major spots suggests that the crystallized NbO_2_ had the distorted rutile structure. At 800 °C, the NbO_2_ film had fully crystallized. The BF image in Fig. 2g shows the NbO_2_ grains imaged along [001] direction. The distorted rutile structure was further confirmed by the SAED pattern from the [001] zone in Fig. 2 h.

**Figure 3:**
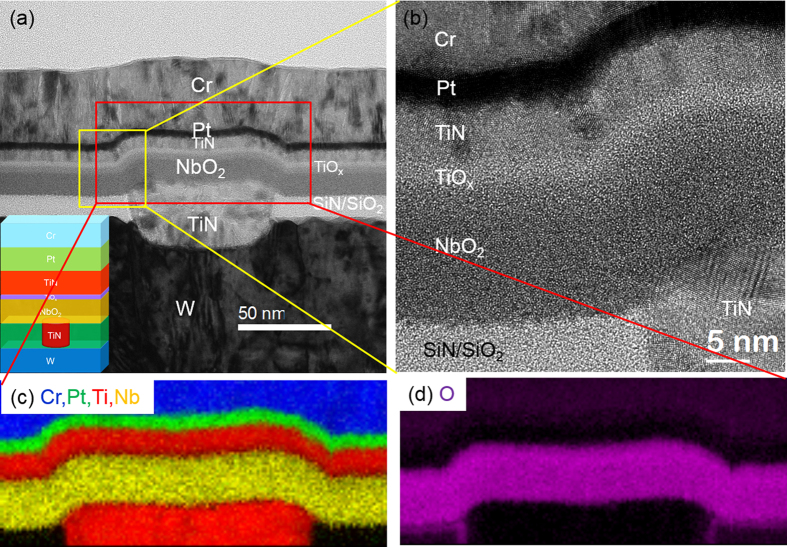
(**a**) Cross-sectional BF TEM image of NbO_2_ MIM device with Cr/Pt/TiN top electrode and TiN/W bottom electrode; the material stacking is shown in the inset. (**b**) High resolution TEM image shows the microstructure of the stacks. Elemental color mapping from STEM/EELS spectrum image shows the composition of Cr, Pt, Ti, and Nb in (**c**) and O in (**d**).

Figure 3 shows an as-prepared 15 nm NbO_2_ layer sandwiched between a Cr/Pt/TiN top electrode and a TiN (nanovia)/W bottom electrode for the MIM device. The NbO_2_ layer had uniform contrast, indicating amorphous structure, which was distinct from metallic electrode materials including W, TiN, Pt, and Cr, all showing crystalline features from diffraction contrast. The thin layer of TiO_x_ (3 nm) was deposited to enhance the adhesion between the top TiN and NbO_2_. High resolution TEM image in Fig. 3b confirmed the amorphous structure in the NbO_2_ and TiO_x_ layers. All the layers maintained their chemical identity without obvious intermixing in the as-prepared state. The color elemental mappings processed from STEM/EELS SI in Fig. 3c,d show the spatial distribution of the elements with the intensity of each color proportional to the corresponding elemental percentage in the material stacks. Figure 3c shows the films of Cr, Pt, TiN, TiO_x_, and NbO_2_ were consistent with the nominal deposition thicknesses. EELS analysis shows that there is no intermixing between Ti and Nb elements. After the TiO_x_ layer was deposited on top of the NbO_2_, a thin oxygen rich layer formed on the top interface of NbO_2_ which probably was caused by the oxidation environment during the deposition of TiO_x_. The O map (Fig. 3d) shows that the thickness of the O layer matches with the sum of the TiO_x_ and NbO_2_ layers. To quantify the composition in the NbO_2_ layer, a middle region of this layer was selected to avoid the rough interface. The EELS quantification showed 35.3 ± 1 at.% Nb in the nominal NbO_2_ layer, with slightly more Nb than the stoichiometric value of 33.3%. No Ti was found in the NbO_2_ layer.

**Figure 4:**
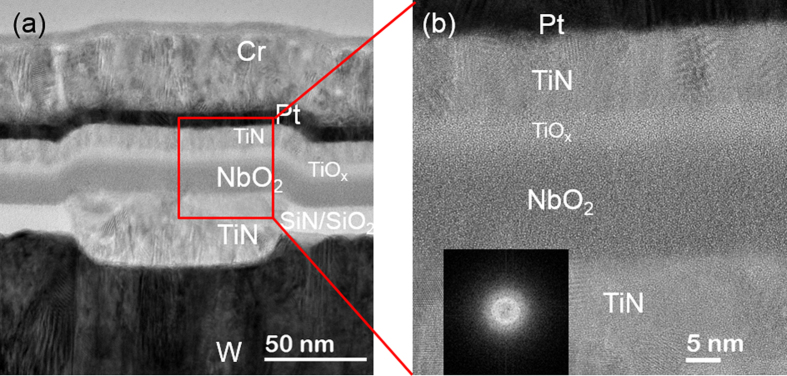
(**a**) Cross-sectional BF TEM image of NbO_2_ MIM device annealed at 650 °C for 1 hr. (**b**) High resolution TEM image shows the microstructure of the stacks.

To study the thermal behavior of nanoscale NbO_2_ in MIM devices, the structure and composition were compared before and after annealing. The amorphous-to-crystalline transition in the nanoscale NbO_2_ sample were found to occur at 700 °C, which was 50 °C lower than that of the 180 nm thick NbO_2_ film. The 15 nm NbO_2_ thin film remained amorphous structure when annealed at 650 °C for 20 min and 1 hr. Figure 4 shows the low magnification TEM image and high resolution image of the 15 nm NbO_2_ thin film device annealed at 650 °C for 1 hr. The NbO_2_ thin film remains amorphous and the NbO_2_/TiO_x_/TiN interfaces are as sharp as the as-fabricated device in Fig. 3. EELS analysis shows that the stoichiometry of the NbO_2_ thin film in the MIM device has 34.4 ± 0.4 at.% Nb upon annealing at 650 °C for 1 hr, close to that of as-fabricated device within experimental error.

**Figure 5:**
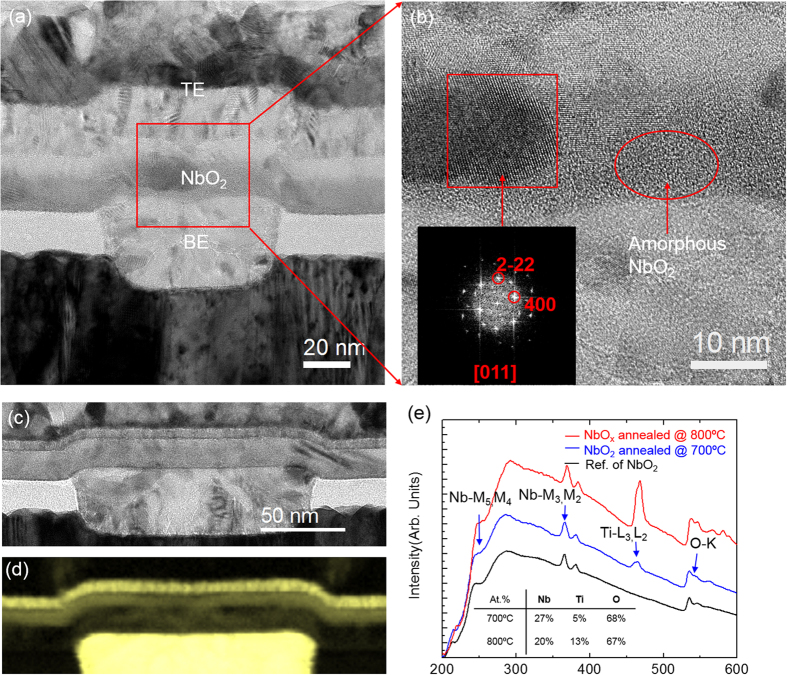
(**a**) Cross-sectional TEM image shows a NbO_2_ MIM device after annealed at 700 °C; (**b**) A HRTEM image zoomed in from the square region in (**a**) shows the NbO_2_ crystalline lattice fringes and amorphous region coexist in the NbO_2_ layer. The inset FFT shows the lattice can be indexed with tetragonal NbO_2_ [011] zone. (**c**) Cross-sectional TEM image of a NbO_2_ device after annealed at 800 °C and (**d**) corresponding Titanium elemental map. (**e**) Ti were observed in the EELS spectra of the NbO_2_ after annealed at 700 and 800 °C with compositions listed in the inset.

Figure 5a shows a TEM image of a NbO_2_ based device after annealing at 700 °C for 20 min. There is crystalline contrast; the bright and dark regions in the NbO_2_ layer are caused by variations in electron scattering due to the orientation of the nanoscale grains. It was also observed that the NbO_2_/TiO_x_/TiN interfaces were not as sharp as in the as-prepared device (Fig. 3), indicating some degree of intermixing between the layers occurred upon annealing at high temperature. The HRTEM image in Fig. 4b shows one of the NbO_2_ crystalline grains with lattice fringes that can be indexed to the distorted rutile structure of NbO_2_ imaged along the [011] zone, as shown in the FFT inset. The thermally induced crystal structure in the 15-nm NbO_2_ layer was the distorted rutile structure, which was consistent with that of the thick NbO_2_ film. In addition, an amorphous region was observed and highlighted in the NbO_2_ layer in the image of Fig. 5b, which indicated 700 °C was a transition temperature from amorphous to crystalline phases. At 800 °C, the NbO_2_ layer was fully crystallized. Furthermore, the EELS spectra and maps (Fig. 5d,e) show that Ti was present in the NbO_2_ layers with increased concentration at higher annealing temperature. However, the composition listed in Fig. 5e may not be accurate since the result was not calibrated against a reference sample with Ti mixed in NbO_2_ thin film. Interpreting the result of the interaction between the NbO_2_ layer and the electrodes at high temperature can be complicated, as multiple valencies of Ti from the TiO_x_ and TiN can evolve during the solid state reaction^21^. Further study of the electrode effects on NbO_2_ during processing are needed.

This study provides a detailed structural and chemical analysis of the evolution of NbO_2_ due to slow external thermal annealing, which differs from the dynamic Joule heating that occurs during a device forming process during short time period (as little as a few nanoseconds) inside the device stack^22^,^23^. However, the observed transition temperature of 700 °C for NbO_2_ crystallization in the MIM device provides a reference point for the calibration of the local temperature inside the device stack under Joule heating during electrically induced forming. The interaction between the switching oxide and the electrodes can change the local chemical composition during forming and subsequent operation, which provides important guidance for the design of selectors and the procedures used to form and operate them^24^,^25^.

In summary, as-fabricated NbO_2_ thin films made by RF magnetron sputtering showed amorphous structure with uniform composition. EELS quantification analysis confirmed that the Nb at.% was 33.7 ± 0.4% in a 180 nm thick film (close to the stoichiometric NbO_2_ composition), and 35.3 ± 1% in a thin NbO_2_ (15 nm) layer in nanoscale MIM devices. In a cross-sectional TEM study, gradual crystallization to a distorted rutile structure was observed at 700-800 °C when thermally annealed in vacuum. Crystallization in the 180 nm NbO_2_ film started at 750 °C with some amorphous regions remaining, and full crystallization occurred upon annealing to 800 °C. Crystallization in the 15 nm NbO_2_ film within the nanoscale MIM devices started at 700 °C, with significant Ti mixed into the Nb oxide layer when annealed at 700–800 °C. These results suggest that crystallization and intermixing with TiN electrodes may occur in nano-scaled Nb oxide layers during operation of NbO_2_-based MIM switching devices, particularly during the high local temperature that typically occur during electrically induced forming step. Because Joule heating underpins the physical mechanism of the NDR behavior^8^, local structural and compositional changes at elevated temperatures should be considered when designing MIM structures for selector applications.

## Methods

Two forms of niobium oxide films (NbO_2_ or Nb_2_O_5_) were deposited by RF magnetron sputtering from either NbO_2_ or Nb_2_O_5_ targets. The deposition chamber base pressure was below 1 × 10^−6^ Torr and the substrates were heated to 450 °C during the deposition. For the 180 nm samples, the NbO_2_ blanket films were deposited directly onto silicon dioxide covered silicon substrates, whereas the 15 nm NbO_2_ thin films were deposited on a nanovia MIM device test platform. To fabricate the devices, a blanket W layer was first deposited by chemical vapor deposition (CVD) on SiO_2_ covered silicon wafer substrates. Then an insulating dielectric layer of silicon nitride/SiO_2_ was deposited on top of the wafers and was subsequently patterned and etched into contact holes of various diameter sizes by the photolithography process. To make the nanovia contacts to the bottom tungsten electrode, a titanium nitride (TiN) film was deposited inside the holes followed by chemical-mechanical polishing. The MIM devices were completed with a top electrode that consisted of 3 nm TiO_x_ (for adhesion purpose), 6 nm TiN, 5 nm Pt, and 40 nm Cr that were deposited sequentially over the NbO_2_ film through a shadow mask. Thermal annealing of the samples was performed in a vacuum chamber at 1 × 10^−6^ Torr between 650 °C and 800 °C for different durations, i.e., 20 minutes and 1 hour. Cross-sectional TEM samples were prepared using a FEI Helios dual beam system. The TEM and EELS characterization were carried out by using a FEI Tecnai equipped with a Gatan GIF Quantum system. Two-dimensional STEM/EELS maps with EELS spectra in the 3^rd^ dimension corresponding to each scanning pixel, called the spectrum image (SI), were collected with a probe size of 0.5 nm at a step resolution of 1 nm.

## Additional Information

**How to cite this article**: Zhang, J. *et al*. Thermally induced crystallization in NbO_2_ thin films. *Sci. Rep.* **6**, 34294; doi: 10.1038/srep34294 (2016).
